# The Role of Vascular-Immune Interactions in Modulating Chemotherapy Induced Neuropathic Pain

**DOI:** 10.3389/fphar.2022.887608

**Published:** 2022-06-22

**Authors:** Tameille Valentine, Lydia Hardowar, Jasmine Elphick-Ross, Richard P. Hulse, Mark Paul-Clark

**Affiliations:** Department of Biosciences, School of Science and Technology, Nottingham Trent University, Nottingham, United Kingdom

**Keywords:** chemotherapy, neuropathy, vascular, permeability, inflammation

## Abstract

Chemotherapy causes sensory disturbances in cancer patients that results in neuropathies and pain. As cancer survivorships has dramatically increased over the past 10 years, pain management of these patients is becoming clinically more important. Current analgesic strategies are mainly ineffective and long-term use is associated with severe side effects. The issue being that common analgesic strategies are based on ubiquitous pain mediator pathways, so when applied to clinically diverse neuropathic pain and neurological conditions, are unsuccessful. This is principally due to the lack of understanding of the driving forces that lead to chemotherapy induced neuropathies. It is well documented that chemotherapy causes sensory neurodegeneration through axonal atrophy and intraepidermal fibre degeneration causing alterations in pain perception. Despite the neuropathological alterations associated with chemotherapy-induced neuropathic pain being extensively researched, underlying causes remain elusive. Resent evidence from patient and rodent studies have indicated a prominent inflammatory cell component in the peripheral sensory nervous system in effected areas post chemotherapeutic treatment. This is accompanied by modulation of auxiliary cells of the dorsal root ganglia sensory neurons such as activation of satellite glia and capillary dysfunction. The presence of a neuroinflammatory component was supported by transcriptomic analysis of dorsal root ganglia taken from mice treated with common chemotherapy agents. With key inflammatory mediators identified, having potent immunoregulatory effects that directly influences nociception. We aim to evaluate the current understanding of these immune-neuronal interactions across different cancer therapy drug classes. In the belief this may lead to better pain management approaches for cancer survivors.

## Introduction

The continued development of novel therapeutics, and improved diagnostic capabilities have resulted in drastic improvements in cancer treatment and survival. In 2020, 3 million people were living with cancer in the United Kingdom, and it is predicted that this number will rise to 5.3 million by 2040 (Maddams et al., 2012). This has been accompanied by a corresponding increase in life expectancy from one year in the early 1970s to 10 years in 2011 (Quaresma et al., 2015). This means that morbidity has become an increasingly important criteria of survivorship. With this in mind, the ubiquitous nature of cancer treatments, which not only target malignant cells, but also have significant impact on the physiological and cellular systems of healthy cells, is becoming more an issue. It is therefore not surprising that cancer treatment results in several long-term side effects that adversely affect the patients’ health. In fact, it has become a prevalent problem, with 68.1% of patients suffering from chemotherapy-induced peripheral neuropathy (CIPN) post-treatment. CIPN encompasses damage to the nervous system attributable for motor function and autonomic control as well as sensory disturbances (Paice, 2011). These sensory disturbances, which include sensory ataxia, painful paraesthesia, and ongoing pins and needles in limb extremities (also known as stocking-glove pattern) is associated with metabolic abnormalities in sensory axons (Seretny et al., 2014; Maihöfner et al., 2021). This has longitudinal consequences as well, with over 30% of patients still experiencing the distress of CIPN 6 months plus after completing chemotherapy cycles (Paice, 2011; Seretny et al., 2014). This debilitating clinical scenario is not just limited to adults, as there is increasing awareness of long-lasting neuropathic pain in survivors of childhood cancer (Lu et al., 2011; Alberts et al., 2018). The accumulating evidence that CIPN is a significant clinical problem, is highlighted by the fact that there is currently no appropriate analgesic treatment for this condition (Haberberger et al., 2019; Guo et al., 2021), with current pain relief strategies (Paice, 2009) being of little benefit (Paice, 2011). There is a small proportion of cancer survivors helped in the short-term by present-day analgesics however, long-term usage is associated with noteworthy adverse complications (Paice, 2019). The ineffectiveness of current treatments highlights our lack of comprehension underpinning mechanisms that result in CIPN development and pathology. It is therefore something that warrants immediate attention and further experimental investigation to help direct novel drug discovery programs for CIPN management, and hopefully prevention. In this perspective, we focus on the inflammatory-driven pathways induced by chemotherapy that led to perturbations in microvascular homeostasis and integrity. As minimal attention has focused on how chemotherapy-induced systemic inflammation causes profound vascular adaptations which drive sensory neurotoxicity.

## Chemotherapy Induced Neuropathic Pain

It is well established that there is an extensive panel of chemotherapeutic agents that causes damage to the peripheral nervous system leading to CIPN symptoms, although their mechanisms of action are notably different (Brissot and Loréal, 2016; Alberts et al., 2018; Branca et al., 2018). Platinum based drugs interact with DNA to form platinum-DNA adducts which induce cellular apoptosis (Beggs et al., 2010; Bodiga et al., 2020), In contrast, vincristine and paclitaxel impair microtubule formation by promoting their instability leading to mitotic arrest and cell death (Flatters and Bennett, 2006; Beggs et al., 2010; Staff et al., 2019; Fumagalli et al., 2021). Whereas Bortezomib acts as a proteasome inhibitor and microtubule destabiliser (Greenlee et al., 2017; Gu et al., 2020). However, all are associated with peripheral hyperalgesia. Over the past decade, research into CIPN has also identified that dorsal root ganglia of sensory neurons are particularly susceptible to platinum-based therapy induced neurotoxicity (Drakesmith et al., 2015; Domoto et al., 2021). Common symptoms of sensory neuropathy follow a similar trajectory of events, initially sensory nerve damage is noted and is accepted to be a precursor to nociceptor sensitization (Tanner et al., 1998). This is accompanied by exaggerated pain responses and ongoing neuronal hyperexcitation which manifests itself as tingling sensations in the hands and feet and chronic pain (Paice, 2009). This progresses to sensory numbness in the extremities, due, at least in part, to intraepidermal sensory nerve fibre regression in the skin (Bosanac et al., 2021). The severity of CIPN experienced by many patients is also responsible for the dose-limiting profile of these chemotherapies and can in some cases lead to cessation of the cancer treatment (Akman et al., 2015; Brissot and Loréal, 2016; Cataldo et al., 2019). This is of increasing clinical concern, as suboptimal treatment of cancer clearly has its own ramifications (Costigan et al., 2009; Brissot and Loréal, 2016; Demaria, 2017).

## Inflammation Mediated Chemotherapy Induced Neuropathic Pain

John Hunter (1794), a Scottish surgeon, described inflammation as a salutary process (Hunter and Home Everard, 1794). In fact, it is our bodies protective mechanism against challenge from injury, infection and noxious stimuli. Chemotherapy by its very nature fits into the noxious stimuli category, and often has many off-target effects due to its permissive nature leading to an inflammatory response. Along with the clear positives that the inflammatory process provides there are detrimental consequences of persistent or aggressive inflammation that result in inappropriate wound healing and adaptive changes to cellular metabolism (Dieli-Conwright et al., 2016). This is highlighted clinically, with high doses of glucocorticoids often pre-administrated prior to particularly platinum-based and taxane chemotherapies (Boulanger et al., 2014). Clinically this helps circumnavigate inflammation and vascular toxicity associated with the chronic use of these agents (Herrmann et al., 2016). The high doses of dexamethasone used suggest that these chemotherapeutic agents cause a profound systemic inflammatory response. It is highly likely that this toxin-induced inflammation contributes significantly to CIPN. In fact, it is known that there is an increase in inflammatory cell influx into tissues where neuropathies are experienced (Laumet et al., 2019), alongside hallmarks of vascular damage (Cameron et al., 2016) and acute kidney injury (McSweeney et al., 2021). These characteristics of disease pathology are accompanied by signs of systemic inflammation highlighted by mitochondrial dysfunction (Canta et al., 2015), hypoxia (Kirchmair et al., 2005), oxidative stress (Shim et al., 2019) and altered metabolism in affected tissue (Ludman and Melemedjian, 2019).

Understanding the different mechanisms involved in the pathogenesis of CIPN will assist in the development of novel therapeutic strategies, not only for treatment, but also as a means of prevention. Current focus is centred on sensory neuronal mitochondrial dysfunction (Horowitz and Greenamyre, 2010; Herrmann et al., 2016; Hohmann et al., 2017). As vincristine and paclitaxel induce loss of intraepidermal nerve fibres, specifically Aδ and C fibres, leading to cold and heat allodynia (Hathway et al., 2017). This arises due to mitochondrial dysfunction and oxidative stress in sensory neurons, with the production of reactive oxygen species a causative factor in CIPN development (Jacobs et al., 1976; Hulse et al., 2015; Hu et al., 2018a; Jager et al., 2020). This has led unfortunately to a consensus in opinion that the development of CIPN is mediated directly through sensory neurotoxicity alone (Kanat et al., 2017). Clearly this is an important aspect of CIPN however, the somatosensory nervous system is a heterogenous population of cells that do not function independently as distinct cellular systems but act as a dynamic multi-cellular network that orchestrate the modulation of nociception and pain acuity.

**FIGURE 1:**
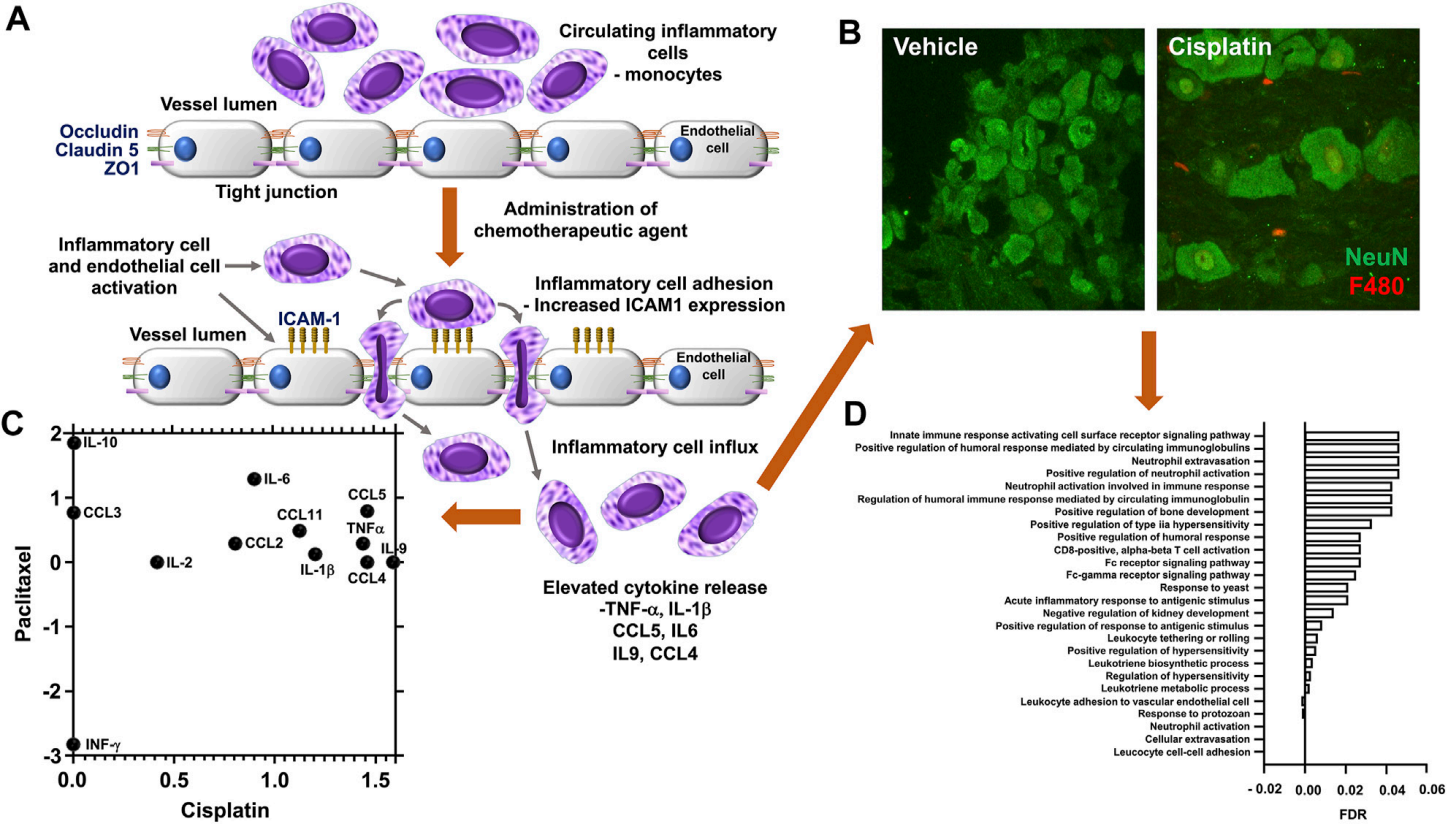
Chemotherapy induced vascular inflammation within mouse dorsal root ganglia **(A)** In rodent models or in human patients administered chemotherapeutic agents display an alternation in the inflammatory profile and vascular permeability of the dorsal root ganglia, factors that accompany the presentation of neuropathic pain behavioural phenotypes following exposure to chemotherapeutic agents such as cisplatin, vincristine or paclitaxel the endothelial cells that form the luminal wall of the capillaries that reside in the dorsal root ganglia have a reduction in tight junctional proteins (ZO1, Occludin, Claudin 5) and accompanying increase in adhesion molecules (ICAM1). This promotes the infiltration of immunological cell types into the dorsal root ganglia and sensory nerves through increased vascular permeability and cell adhesion. **(B)** Representative images of the increased infiltration of macrophages is displayed following biweekly intraperitoneal injection of Cisplatin (ip 2 mg/kg for 3 weeks) to C57bl6 mice versus vehicle control group (unpublished data). Accumulation of F4/80 positive inflammatory cells (Red) when compared to sham treated rodents, in close apposition to the sensory neurons (green NeuN) in the DRG. In addition, **(C)** Chemotherapy induced sensory neuroinflammation is represented by the pronounced proinflammatory environment of the peripheral sensory nervous system. To highlight this, here we present the comparison of available rodent dorsal root ganglia transcriptome datasets, obtained from Gene Expression Omnibus (GEO) following either cisplatin [GSE125003 (Starobova et al., 2020)] or Paclitaxel [GSE185084 (Lessans et al., 2019)] administration, comparing log 2-fold change of Fragments Per Kilobase Million (FPKM) values from vehicle controls. Our reanalysis of this data demonstrates an increase in proinflammatory mediator expression in particular IL6. **(D)** Furthermore, utilising FPKM values to summarise the transcriptome following cisplatin treatment from two independent rodent studies [GSE64174 (unpublished) and GSE125003 (Starobova et al., 2020)] highlights an alteration in the dorsal root ganglia transcriptome profile. In both studies797 genes were identified with altered gene expression (FPKM value) greater than 1 log fold change from controls samples). Additionally, using gene enrichment analysis (STRING) post cisplatin treatment the identified 797 genes had a strong association with the promotion of pro-inflammatory process [Gene Ontology Biological Process of less than 0.5 Log *p*-value with False Discovery Rate (FDR)].

There is increasing evidence that sensory neuroinflammation is a key component of this CIPN development (Fumagalli et al., 2021), and it is now widely appreciated that neuropathic pain originates from sensory neuron damage caused by these agents. In fact, for platinum-based therapies, which account for 50–70% of cancer treatments, neurotoxicity as previously highlighted is the major side effect (Calls et al., 2020). Most work in this area has centred on DRGs as the source material. DRGs are a heterogeneous tissue, that not only contain the cell bodies of sensory nerves, but also incorporate endothelial cells, fibroblasts, satellite glial cells, and immune cells. A recent transcriptomic study in rodents treated with platinum-based therapies and vincristine showed a significant enrichment of genes that are associated with bone marrow derived cells and granulocytes (Starobova et al., 2020). The effects of administering these agents in a number of studies have been shown to elevated systemic levels of key proinflammatory cytokines that include TNF-α (Akman et al., 2015), IL-1β (Ma et al., 2022), IL-6 and IL-8 (Kiss et al., 2020; Laino et al., 2020), Figures 1A,C and a decrease in the tissue protective activity of T-reg/Th17 cells (Li et al., 2016; Wan et al., 2016). This pro-inflammatory environment observed in DRG’s is clearly highlighted in two transcriptomic rodent studies represented in Figure 1C where clear similarities in the profiles of effected genes, with 6.5 K genes similarly differentially expressed after platinum-based therapy exposure (Lessans et al., 2019; Starobova et al., 2020). Interestingly, clinically there is a direct correlation in circulating levels of acute inflammatory cytokines IL-6, IL-8 and CCL2 and the magnitude of perceived pain after surgery (Wang et al., 2009). Suggesting that these cytokines that are elicited by chemotherapeutic agents have direct impact on neuropathic pain.

Some discrepancies in the literature are evident. These can be attributed to the time that measurements were taken. As most *in vivo* studies have focused on the pain element of the experiment, which takes between 1 and 2 weeks to develop. Therefore, often peaks in the systemic inflammatory response to these agents have subsided, however hallmarks of the process remain evident within the CIPN affected tissue. This is apparent from data generated in our lab and that of others (Figure 1). Analysis of gene expression data from a similar model revealed profound changes in immune response, regulation of cellular transport, neuronal and DNA/RNA repair processes (Starobova et al., 2020). In these murine cisplatin-induced neuropathy models, there is clear immunocytochemical staining evidence of neutrophil and monocyte influx within the DRGs (Figures 1B,D). There are also clear footprints of several inflammatory processes which include, hypoxia, oxidative stress and altered cellular metabolism. As a high number of genes that were highly deferentially expressed in the DRG tissue were associated with hypoxia, oxidative stress and mitochondrial metabolism as identified by networks and pathways analysis (STRING) of differentially expressed within the two data sets.

Systemic inflammation caused by chemotherapy is known to cause lesions within the somatosensory nervous system. This inflammation influences not only nociceptors but also neighbouring supportive cellular systems, leading to maladaptive plasticity within the nociceptive system (Costigan et al., 2009). This along with increased satellite glia expression of IL-1β, results in changes to vascular permeability (Montague-Cardoso et al., 2020). These mechanisms both contribute to CIPN. However, in several studies immune activity is not recognised (Makker et al., 2017). As a comparative study of paclitaxel and oxaliplatin in rodents, although pronounced systemic inflammation was evident there was a lack of inflammatory cell infiltration into sensory tissues. Further, depletion of regulatory T-cells in this study failed to impact upon chemotherapy-induced pain behaviours (Makker et al., 2017). There are also several studies concluding that chemotherapy induced sensory neuropathy is peripherally restricted due to increased accumulation of chemotherapeutic agents in the PNS (Ta et al., 2006). However, this conclusion ignores several key factors and makes several assumptions. It doesn’t consider activation of resident inflammatory cell already present within PNS tissue and the influence that cytokines produced by these cells has on nociception (Domoto et al., 2021). Also, models used in these studies utilise many differing rodent species and strains (Marmiroli et al., 2017), which could possibly relate to the clinical demographic that accompanies CIPN susceptibility (Greenlee et al., 2017). Similarly, differences in rodent sex can also be implicated in the discrepancies observed in CIPN outcomes (Luo et al., 2019; Legakis et al., 2020; Saika et al., 2020). This highlights the requirement for the alignment of standardised rodent models, delivery routes, dosing frequencies and regimens to achieve more parity.

## Cellular Mechanisms of Chemotherapy Induced Inflammation

Several classifications of chemotherapy drugs, including platinum-based chemotherapeutic agents, share a common mode of action. They induce cell senescence through several different mechanisms that halt tumour progression and promote cell death. However, many of these agents lack selectivity between cancer cells and healthy host cells. This indiscriminate nature of chemotherapies forms the major basis of side-effect profiles attributed to these treatments (Demaria, 2017). As discussed, the toxic nature of cancer therapy induces a profound inflammatory response in the circulation, CNS and PNS, resulting in many disparate comorbidities that include innate and adaptive cell activation, neuropathies, and fatigue (Vichaya et al., 2015). Alongside these sensory disturbances, a common side-effect of many chemotherapy treatments that displays similarities in vascular mediated inflammation in relation is nephrotoxicity. Chemotherapy-induced kidney dysfunction has been linked to elevated circulating levels of IL-18, IL-1β, IL-6 and IL-33 (Miyagi et al., 2014; Nozaki et al., 2015; Ravichandran et al., 2017), and is reflective of what is occurring systemically with respect to inflammation. This clearly has repercussions on metabolism and cell signalling pathways (Portilla et al., 2006), cumulating in a detrimental impact on survivor morbidity.

CIPN induction is dependent upon macrophage infiltration, as macrophage depletion and suppression of monocyte chemotactic protein 1 (MCP1) inhibits CIPN development (Zhang et al., 2016). The presence of inflammatory cells in somatosensory tissues are accompanied by the release of a diverse array of growth factors and cytokines that can influence nociceptive circuits. These proinflammatory mediators are known to stimulate signalling cascades, which have been shown to heighten activity of fundamental ion channels and known to be implicated in nociceptor activity (i.e., activation of Transient Receptor Potential Vanilloid 1 (TRPV1) (Kuai et al., 2020) and Transient Receptor Potential Ankryin 1 (TRPA1) (Old et al., 2014). Vincristine activated CX3CR1 macrophages through reactive oxygen species, resulting in release of cytokines known to stimulate TRPA1 in peripheral neurons (Old et al., 2014; Montague et al., 2018). Cisplatin-induced prostaglandin E2 production drives TRPV1 activity (Poulsen et al., 2014), whilst oxaliplatin induced CIPN through Toll like Receptor 4 driven matrix metalloproteinase 9/2 production from macrophages and neurons (Gu et al., 2020). Other studies show a correlative link (Figure 1) between levels of TNFα (Liu et al., 2019), CXCL1 (Luo et al., 2019), IL-1β and IL-6 (Wan et al., 2016; Abdelsameea and Kabil, 2018; Onk et al., 2018) and nociceptor activation (Black et al., 2018). Conversely, the addition or presence of anti-inflammatory cytokines, such as IL-10 can suppress neuropathic pain (McKelvey et al., 2015) and nociceptor activation. This is due to a dampening of nociceptor excitability and leading to a faster recovery from CIPN (Krukowski et al., 2016). Satellite glia in the DRGs is also implicated in neuropathic pain development, alongside migrating of immune cells into PNS tissues with the maintenance and longevity of neuropathic pain (Jager et al., 2020).

A major contributor to the systemic inflammatory response is IL-6, often the predominant cytokine measured in chronic disease states and is associated by premature aging of numerous systems (Maggio et al., 2006). Interestingly, elevated levels of this cytokine have been measured in patients suffering from CIPN (Kiss et al., 2020) and in spinal cord microglia from rodent studies (Cataldo et al., 2019). Elevated IL-6 is known to induce local levels of hepcidin, which intern can downregulate ferroportin, the key regulator of free iron transporter in and out of the cell (Drakesmith et al., 2015). Transient levels of IL-6 and hepcidin may act in regulating homeostatic iron transport within cells, however persistent elevated levels of IL-6 induced hepcidin would result in excess intracellular iron and iron-related toxicity (Brissot and Loréal, 2016; Muckenthaler et al., 2017). This occurs as ferroportin is the exclusive transporter of free iron in and out of the cells particularly in macrophages, which are an important cellular store of iron. Most of the bodies iron is recycled (90%) and not absorbed therefore this process of free movement of iron in and out of macrophages plays an important part in controlling iron homeostasis. Down regulation of ferroportin in macrophages exerts control of iron’s distribution within tissues. This occurs as part innate immune response to infection as a critical mechanism in withholding iron from bacterial pathogens and maintaining relatively low iron conditions inhibiting microbial growth (Prentice, 2017). Excessive iron within cells is known to increase cellular damage through elevates ROS production catalysed by the Fenton reaction (Galaris et al., 2019). This disturbance in the redox balance would also disrupt mitochondrial function (Walter et al., 2002) and effects macrophage polarisation (Agoro et al., 2018). This disruption in iron homeostasis has also been linked to anaemia of chronic disease, where circulating IL-6 levels are elevated (Madu and Ughasoro, 2017). In patients suffering with hereditary hemochromatosis, where you get systemic iron overload, peripheral neuropathies are common (Piperno et al., 2020). However, although there is evidence that hepcidin and ferroportin are important in central brain iron homeostasis (Vela, 2018), little is known about the PNS (Levi and Taveggia, 2014). Reanalysis of the transcriptomic data from the two DRG murine CIPN models shows a significant downregulation of ferroportin in both studies, implicating iron handling in the onset of chemotherapy-induced inflammation (Lessans et al., 2019; Starobova et al., 2020). This, if sustained would cause excessive cellular iron overload, leading to elevated ROS production through the Fenton reaction, mitochondrial dysfunction and altered neuronal activity (Horowitz and Greenamyre, 2010). Furthermore, inhibition of iron-induced ferroptosis inhibited neuropathic pain reactions in peripheral nerves (Guo et al., 2021). The consequences of iron toxicity and not just relevant to neurons but have profound effects on supporting tissue. Excessive iron loading in endothelial cells induces cholesterol synthesis and also makes them more susceptible to TNFα induced apoptosis (Fisher et al., 2021). This would have the functional consequence of increasing hypoxia within the CIPN affected area as DRGs are highly vascularised (Jacobs et al., 1976a; Kiernan, 1996). Iron dysregulation in macrophages also impacts on this cells metabolic and inflammatory profile increased TNFα and NO expression while suppressing the anti-inflammatory cytokine expression of IL-10 (Cronin et al., 2019). This again would promote the inflammatory cycle further increase damage to neurons and endothelial cell within the DRG. The mechanism by which iron induces damage is still highly speculative but has been suggested being through TGFβ signalling and autophagy. Evidence of this is present within the global profiling of the two DRG studies with SMAD1 and 2 (TGFβ signalling) and ATG3 (autophagy) upregulated in both studies (Lessans et al., 2019; Starobova et al., 2020). With both mechanisms being implicated in CIPN (Fukuda et al., 2017; Kim et al., 2020).

There is therefore clear evidence that inflammation and its consequences play an integral part of CIPN. Interestingly, glucocorticoids are used clinically as the most effective strategy against inflammatory pain with a possible role on every step of nociception. There is unfortunately little evidence for their effectiveness on CIPN in patients and a lack of parity in animal models (Leppert and Buss, 2012).

## Disturbances in Vascular Permeability Within the Somatosensory System

Dorsal root ganglion are susceptible to the accumulation of pharmacological agents, which include chemotherapeutic agents (platinum compounds). This is due to an endothelial barrier that provides minimal protection (Allen and Kiernan, 1994; Ta et al., 2006; Podratz et al., 2011). Several studies have characterised the DRG capillary network indicating a highly vascularized sensory tissue. The DRG capillaries are highly fenestrated in appearance resulting in high levels of vascular permeability (Jacobs et al., 1976; Kiernan, 1996), a process exacerbated during sensory neuropathology (Hulse et al., 2015). However, there are disparities in relation to other vascular neural beds, as the nerve trunk has a markedly reduced capillary network in comparison (Jimenez-Andrade et al., 2008; Haberberger et al., 2019). Whereas the blood brain and blood spinal cord barriers are largely impermeable under normal physiological conditions (Beggs et al., 2010). Therefore, there is an abundant accumulation of the chemotherapeutic agents in the DRG post-administration (Meijer et al., 1999; McDonald et al., 2005) in comparison to other tissues including the central nervous system, in particular brain and spinal cord (McDonald et al., 2005). As a result of this susceptibility to toxic agents penetrating the PNS, sensory complications in rodents (Vencappa et al., 2015; Schappacher et al., 2017; Hu et al., 2018b) and humans (Krarup-Hansen et al., 2007; Staff et al., 2019) are accompanied by degeneration of the peripheral sensory nervous system. Hallmarks of sensory neurodegeneration include diminished sensory nerve conduction and reduced intraepidermal nerve fibre density following administration of platinum-based chemotherapy (Hathway et al., 2017), Taxols (Flatters and Bennett, 2006) or vinca alkaloids (Boehmerle et al., 2014). This is exacerbated by the fact the endothelium that forms the lumen of the dorsal root ganglion capillaries are also susceptible to chemotherapy exposure (Hohmann et al., 2017b). Cisplatin directly causes endothelial cell dysfunction by reducing the expression of vasodilator nitric oxide (NO) *in vitro* and is associated with increased intima-media thickening in patients (Sekijima et al., 2011). In line with increased PNS neuroinflammation in CIPN, platinum-based agents induce reductions in tight junctional protein expression of Zona Occludin1 (ZO-1) (Branca et al., 2018) and reduced transendothelial electrical resistance (Bodiga et al., 2015). This results in increased vascular permeability facilitating transmigration of inflammatory cell types across the endothelial cell wall (Bodiga et al., 2015). Furthermore, cisplatin (Nuver et al., 2010; Bodiga et al., 2015) and vincristine (Old et al., 2014) elevate Intercellular Adhesion Molecule 1 (ICAM-1) expression (as summarised in Figure 1) in endothelial cells alongside enhancing Akt signalling (Bodiga et al., 2020), in an NF kappa B dependent manner (Yu et al., 2008). This initiates inflammatory cell adhesion to the endothelium promoting trafficking and infiltration of these cells into peripheral sensory tissues. This disturbance to capillary function and integrity is mirrored in other physiological systems, with alterations in blood flow occurring in cisplatin-induced rodent models of renal failure (Winston and Safirstein, 1985). Toll-like 4 receptor (TLR4) is an important innate immune receptor involved in the inflammatory response. Mice deficient in TLR4 demonstrate reduced accumulation of leukocytes in cisplatin-induced kidney injury, which was accompanied by ameliorated levels of inflammatory serum marker (West et al., 2006; Ramesh et al., 2007; Zhang et al., 2008; Summers et al., 2011). This evidence contributes to the evolving fundamental importance of the vasculature within the somatosensory nervous system in promoting a pro-inflammatory environment responsible for the development of CIPN. This is supported by a growing body of data showing extensive infiltration of circulatory inflammatory cells into the peripheral sensory nerve trunks and dorsal root ganglion. This highlights the importance of putative inflammatory derived mechanisms [CX3CR1 mediated (Old et al., 2014; Montague et al., 2018), TLR4 (Starobova et al., 2019)] attributable to the onset of CIPN and supported by a recent study where in a vincristine rodent model of CIPN increased adhesion and infiltration of CCR_2_ positive inflammatory monocytes were seen. This was accompanied by reductions in endothelial tight junction proteins (Claudin 5, ZO1), elevated levels of ICAM-1 and vascular leakage which paralleled neuropathic pain development (Montague-Cardoso et al., 2020).

## Discussion

Here we provide evidence for pronounced pro-inflammatory dependent activation of nociceptor function in CIPN development. We highlight the fact that recent studies acknowledge the essential role the capillary network has in facilitating chemotherapy induced pro-inflammatory processes. Here we provide strong cohesive pathological evidence for the role of inflammation and the microvasculature within nociceptive tissues as significant components in CIPN development.

## Data Availability

Publicly available datasets were analyzed in this study. This data can be found here: GSE125003, GSE185084, GSE64174.

## References

[B1] Abdelsameea A. A. Kabil S. L. (2018). Mitigation of Cisplatin-Induced Peripheral Neuropathy by Canagliflozin in Rats. Naunyn Schmiedeb. Arch. Pharmacol. 391, 945–952. 10.1007/s00210-018-1521-5 29862426

[B2] Agoro R. Taleb M. Quesniaux V. F. J. Mura C. (2018). Cell Iron Status Influences Macrophage Polarization. PLOS ONE 13, e0196921. 10.1371/journal.pone.0196921 29771935 PMC5957380

[B3] Akman T. Akman L. Erbas O. Terek M. C. Taskiran D. Ozsaran A. (20152015). The Preventive Effect of Oxytocin to Cisplatin-Induced Neurotoxicity: An Experimental Rat Model. Biomed. Res. Int. 2015, 167235–5. 10.1155/2015/167235 PMC4320931 25688351

[B4] Alberts N. M. Gagnon M. M. Stinson J. N. (2018). Chronic Pain in Survivors of Childhood Cancer: a Developmental Model of Pain across the Cancer Trajectory. Pain 159, 1916–1927. 10.1097/j.pain.0000000000001261 29708940

[B5] Allen D. T. Kiernan J. A. (1994). Permeation of Proteins from the Blood into Peripheral Nerves and Ganglia. Neuroscience 59, 755–764. 10.1016/0306-4522(94)90192-9 8008217

[B6] Beggs S. Liu X. J. Kwan C. Salter M. W. (2010). Peripheral Nerve Injury and TRPV1-Expressing Primary Afferent C-Fibers Cause Opening of the Blood-Brain Barrier. Mol. Pain 6, 74–8069. 10.1186/1744-8069-6-74 21044346 PMC2984489

[B7] Black B. J. Atmaramani R. Kumaraju R. Plagens S. Romero-Ortega M. Dussor G. (2018). Adult Mouse Sensory Neurons on Microelectrode Arrays Exhibit Increased Spontaneous and Stimulus-Evoked Activity in the Presence of Interleukin-6. J. Neurophysiol. 120, 1374–1385. 10.1152/jn.00158.2018 29947589 PMC6171072

[B8] Bodiga V. L. Bathula J. Kudle M. R. Vemuri P. K. Bodiga S. (2020). Andrographolide Suppresses Cisplatin-Induced Endothelial Hyperpermeability through Activation of PI3K/Akt and eNOS -derived Nitric Oxide. Bioorg Med. Chem. 28, 115809. 10.1016/j.bmc.2020.115809 33065471

[B9] Bodiga V. L. Kudle M. R. Bodiga S. (2015). Silencing of PKC-α, TRPC1 or NF-Κb Expression Attenuates Cisplatin-Induced ICAM-1 Expression and Endothelial Dysfunction. Biochem. Pharmacol. 98, 78–91. 10.1016/j.bcp.2015.08.101 26300057

[B10] Boehmerle W. Huehnchen P. Peruzzaro S. Balkaya M. Endres M. (2014). Electrophysiological, Behavioral and Histological Characterization of Paclitaxel, Cisplatin, Vincristine and Bortezomib-Induced Neuropathy in C57Bl/6 Mice. Sci. Rep. 4, 6370. 10.1038/srep06370 25231679 PMC5377307

[B11] Bosanac T. Hughes R. O. Engber T. Devraj R. Brearley A. Danker K. (2021). Pharmacological SARM1 Inhibition Protects Axon Structure and Function in Paclitaxel-Induced Peripheral Neuropathy. Brain 144, 3226–3238. 10.1093/brain/awab184 33964142 PMC8634121

[B12] Boulanger J. Boursiquot J. N. Cournoyer G. Lemieux J. Masse M. S. Almanric K. (2014). Management of Hypersensitivity to Platinum- and Taxane-Based Chemotherapy: Cepo Review and Clinical Recommendations. Curr. Oncol. 21, e630–41. 10.3747/co.21.1966 25089112 PMC4117628

[B13] Branca J. J. V. Maresca M. Morucci G. Becatti M. Paternostro F. Gulisano M. (2018). Oxaliplatin-induced Blood Brain Barrier Loosening: a New Point of View on Chemotherapy-Induced Neurotoxicity. Oncotarget 9, 23426–23438. 10.18632/oncotarget.25193 29805744 PMC5955120

[B14] Brissot P. Loréal O. (2016). Iron Metabolism and Related Genetic Diseases: A Cleared Land, Keeping Mysteries. J. Hepatol. 64, 505–515. 10.1016/j.jhep.2015.11.009 26596411

[B15] Calls A. Carozzi V. Navarro X. Monza L. Bruna J. (2020). Pathogenesis of Platinum-Induced Peripheral Neurotoxicity: Insights from Preclinical Studies. Exp. Neurol. 325, 113141. 10.1016/j.expneurol.2019.113141 31865195

[B16] Cameron A. C. Touyz R. M. Lang N. N. (2016). Vascular Complications of Cancer Chemotherapy. Can. J. Cardiol. 32, 852–862. 10.1016/j.cjca.2015.12.023 26968393 PMC4989034

[B17] Canta A. Pozzi E. Carozzi V. A. (2015). Mitochondrial Dysfunction in Chemotherapy-Induced Peripheral Neuropathy (CIPN). Toxics 3, 198–223. 10.3390/toxics3020198 29056658 PMC5634687

[B18] Cataldo G. Erb S. J. Lunzer M. M. Luong N. Akgün E. Portoghese P. S. (2019). The Bivalent Ligand MCC22 Potently Attenuates Hyperalgesia in a Mouse Model of Cisplatin-Evoked Neuropathic Pain without Tolerance or Reward. Neuropharmacology 158, 107598. 10.1016/j.neuropharm.2019.04.004 30970233 PMC6745246

[B19] Costigan M. Scholz J. Woolf C. J. (2009). Neuropathic Pain: A Maladaptive Response of the Nervous System to Damage. Annu. Rev. Neurosci. 32, 1–32. 10.1146/annurev.neuro.051508.135531 19400724 PMC2768555

[B20] Cronin S. J. F. Woolf C. J. Weiss G. Penninger J. M. (2019). The Role of Iron Regulation in Immunometabolism and Immune-Related Disease. Front. Mol. Biosci. 6, 116. 10.3389/fmolb.2019.00116 31824960 PMC6883604

[B21] Demaria M. (2017). Senescent Cells: New Target for an Old Treatment? Mol. Cell. Oncol. 4, e1299666. 10.1080/23723556.2017.1299666 28616578 PMC5462517

[B22] Dieli-Conwright C. M. Wong L. Waliany S. Bernstein L. Salehian B. Mortimer J. E. (2016). An Observational Study to Examine Changes in Metabolic Syndrome Components in Patients with Breast Cancer Receiving Neoadjuvant or Adjuvant Chemotherapy. Cancer 122, 2646–2653. 10.1002/cncr.30104 27219902 PMC4992442

[B23] Domoto R. Sekiguchi F. Tsubota M. Kawabata A. (2021). Macrophage as a Peripheral Pain Regulator. Cells 10, 1881. 10.3390/cells10081881 34440650 PMC8392675

[B24] Drakesmith H. Nemeth E. Ganz T. (2015). Ironing Out Ferroportin. Cell. Metab. 22, 777–787. 10.1016/j.cmet.2015.09.006 26437604 PMC4635047

[B25] Fisher A. L. Srole D. N. Palaskas N. J. Meriwether D. Reddy S. T. Ganz T. (2021). Iron Loading Induces Cholesterol Synthesis and Sensitizes Endothelial Cells to TNFα-Mediated Apoptosis. J. Biol. Chem. 297, 101156. 10.1016/j.jbc.2021.101156 34480898 PMC8463868

[B26] Flatters S. J. Bennett G. J. (2006). Studies of Peripheral Sensory Nerves in Paclitaxel-Induced Painful Peripheral Neuropathy: Evidence for Mitochondrial Dysfunction. Pain 122, 245–257. 10.1016/j.pain.2006.01.037 16530964 PMC1805481

[B27] Fukuda Y. Li Y. Segal R. A. (2017). A Mechanistic Understanding of Axon Degeneration in Chemotherapy-Induced Peripheral Neuropathy. Front. Neurosci. 11, 481. 10.3389/fnins.2017.00481 28912674 PMC5583221

[B28] Fumagalli G. Monza L. Cavaletti G. Rigolio R. Meregalli C. (2020). Neuroinflammatory Process Involved in Different Preclinical Models of Chemotherapy-Induced Peripheral Neuropathy. Front. Immunol. 11, 626687. 10.3389/fimmu.2020.626687 33613570 PMC7890072

[B29] Galaris D. Barbouti A. Pantopoulos K. (2019). Iron Homeostasis and Oxidative Stress: An Intimate Relationship. Biochim. Biophys. Acta Mol. Cell. Res. 1866, 118535. 10.1016/j.bbamcr.2019.118535 31446062

[B30] Greenlee H. Hershman D. L. Shi Z. Kwan M. L. Ergas I. J. Roh J. M. (2017). BMI, Lifestyle Factors and Taxane-Induced Neuropathy in Breast Cancer Patients: The Pathways Study. J. Natl. Cancer Inst. 109, djw206. 10.1093/jnci/djw206 27794123 PMC6093415

[B31] Gu H. Wang C. Li J. Yang Y. Sun W. Jiang C. (2020). High Mobility Group Box-1-toll-like Receptor 4-phosphatidylinositol 3-kinase/protein Kinase B-Mediated Generation of Matrix Metalloproteinase-9 in the Dorsal Root Ganglion Promotes Chemotherapy-Induced Peripheral Neuropathy. Int. J. Cancer 146, 2810–2821. 10.1002/ijc.32652 31465111

[B32] Guo Y. Du J. Xiao C. Xiang P. Deng Y. Hei Z. (2021). Inhibition of Ferroptosis-like Cell Death Attenuates Neuropathic Pain Reactions Induced by Peripheral Nerve Injury in Rats. Eur. J. Pain 25, 1227–1240. 10.1002/ejp.1737 33497529

[B33] Haberberger R. V. Barry C. Dominguez N. Matusica D. (2019). Human Dorsal Root Ganglia. Front. Cell. Neurosci. 13, 271. 10.3389/fncel.2019.00271 31293388 PMC6598622

[B34] Hathway G. J. Murphy E. Lloyd J. Greenspon C. Hulse R. P. (2018). Cancer Chemotherapy in Early Life Significantly Alters the Maturation of Pain Processing. Neuroscience 387, 214–229. 10.1016/j.neuroscience.2017.11.032 29196027 PMC6150930

[B35] Herrmann J. Yang E. H. Iliescu C. A. Cilingiroglu M. Charitakis K. Hakeem A. (2016). Vascular Toxicities of Cancer Therapies: The Old and the New--An Evolving Avenue. Circulation 133, 1272–1289. 10.1161/CIRCULATIONAHA.115.018347 27022039 PMC4817363

[B36] Hohmann S. W. Angioni C. Tunaru S. Lee S. Woolf C. J. Offermanns S. (2017). The G2A Receptor (GPR132) Contributes to Oxaliplatin-Induced Mechanical Pain Hypersensitivity. Sci. Rep. 7, 446. 10.1038/s41598-017-00591-0 28348394 PMC5428564

[B37] Horowitz M. P. Greenamyre J. T. (2010). Mitochondrial Iron Metabolism and its Role in Neurodegeneration. J. Alzheimers Dis. 20 Suppl 2, S551–S568. 10.3233/JAD-2010-100354 20463401 PMC3085540

[B38] Hu L. Y. Zhou Y. Cui W. Q. Hu X. M. Du L. X. Mi W. L. (2018a). Triggering Receptor Expressed on Myeloid Cells 2 (TREM2) Dependent Microglial Activation Promotes Cisplatin-Induced Peripheral Neuropathy in Mice. Brain Behav. Immun. 68, 132–145. 10.1016/j.bbi.2017.10.011 29051087

[B39] Hu L. Y. Zhou Y. Cui W. Q. Hu X. M. Du L. X. Mi W. L. (2018b). Triggering Receptor Expressed on Myeloid Cells 2 (TREM2) Dependent Microglial Activation Promotes Cisplatin-Induced Peripheral Neuropathy in Mice. Brain Behav. Immun. 68, 132–145. 10.1016/j.bbi.2017.10.011 29051087

[B40] Hulse R. P. Beazley-Long N. Ved N. Bestall S. M. Riaz H. Singhal P. (2015). Vascular Endothelial Growth Factor-A165b Prevents Diabetic Neuropathic Pain and Sensory Neuronal Degeneration. Clin. Sci. (Lond) 129, 741–756. 10.1042/CS20150124 26201024

[B41] Hunter J. Home Everard S. (1794). 1728-1793, A Treatise on the Blood, Inflammation, and Gun-Shot Wounds. London: 1756 1832. Printed by John Richardson for George Nicol Available at: http://hdl.handle.net/10713/3049.

[B42] Jacobs J. M. Macfarlane R. M. Cavanagh J. B. (1976). Vascular Leakage in the Dorsal Root Ganglia of the Rat, Studied with Horseradish Peroxidase. J. Neurol. Sci. 29, 95–107. 10.1016/0022-510x(76)90083-6 950578

[B43] Jager S. E. Pallesen L. T. Richner M. Harley P. Hore Z. McMahon S. (2020). Changes in the Transcriptional Fingerprint of Satellite Glial Cells Following Peripheral Nerve Injury. Glia 68, 1375–1395. 10.1002/glia.23785 32045043

[B44] Jimenez-Andrade J. M. Herrera M. B. Ghilardi J. R. Vardanyan M. Melemedjian O. K. Mantyh P. W. (2008). Vascularization of the Dorsal Root Ganglia and Peripheral Nerve of the Mouse: Implications for Chemical-Induced Peripheral Sensory Neuropathies. Mol. Pain 4, 10–18. 10.1186/1744-8069-4-10 18353190 PMC2289805

[B45] Kanat O. Ertas H. Caner B. (2017). Platinum-induced Neurotoxicity: A Review of Possible Mechanisms. World J. Clin. Oncol. 8, 329–335. 10.5306/wjco.v8.i4.329 28848699 PMC5554876

[B46] Kiernan J. A. (1996). Vascular Permeability in the Peripheral Autonomic and Somatic Nervous Systems: Controversial Aspects and Comparisons with the Blood-Brain Barrier. Microsc. Res. Tech. 35, 122–136. 10.1002/(SICI)1097-0029(19961001)35:2<122::AID-JEMT3>3.0.CO;2-S 8923447

[B47] Kim H. K. Lee S. Y. Koike N. Kim E. Wirianto M. Burish M. J. (2020). Circadian Regulation of Chemotherapy-Induced Peripheral Neuropathic Pain and the Underlying Transcriptomic Landscape. Sci. Rep. 10, 13844. 10.1038/s41598-020-70757-w 32796949 PMC7427990

[B48] Kirchmair R. Walter D. H. Ii M. Rittig K. Tietz A. B. Murayama T. (2005). Antiangiogenesis Mediates Cisplatin-Induced Peripheral Neuropathy: Attenuation or Reversal by Local Vascular Endothelial Growth Factor Gene Therapy without Augmenting Tumor Growth. Circulation 111, 2662–2670. 10.1161/CIRCULATIONAHA.104.470849 15897348

[B49] Kiss E. Abdelwahab E. H. M. M. Steib A. Papp E. Torok Z. Jakab L. (2020). Cisplatin Treatment Induced Interleukin 6 and 8 Production Alters Lung Adenocarcinoma Cell Migration in an Oncogenic Mutation Dependent Manner. Respir. Res. 21, 120. 10.1186/s12931-020-01389-x 32434541 PMC7238555

[B50] Krarup-Hansen A. Helweg-Larsen S. Schmalbruch H. Rørth M. Krarup C. (2007). Neuronal Involvement in Cisplatin Neuropathy: Prospective Clinical and Neurophysiological Studies. Brain 130, 1076–1088. 10.1093/brain/awl356 17301082

[B51] Krukowski K. Eijkelkamp N. Laumet G. Hack C. E. Li Y. Dougherty P. M. (2016). CD8+ T Cells and Endogenous IL-10 Are Required for Resolution of Chemotherapy-Induced Neuropathic Pain. J. Neurosci. 36, 11074–11083. 10.1523/JNEUROSCI.3708-15.2016 27798187 PMC5098842

[B52] Kuai C. P. Ju L. J. Hu P. P. Huang F. (2020). Corydalis Saxicola Alkaloids Attenuate Cisplatin-Induced Neuropathic Pain by Reducing Loss of IENF and Blocking TRPV1 Activation. Am. J. Chin. Med. 48, 407–428. 10.1142/S0192415X20500214 32138533

[B53] Laino A. S. Woods D. Vassallo M. Qian X. Tang H. Wind-Rotolo M. (2020). Serum Interleukin-6 and C-Reactive Protein Are Associated with Survival in Melanoma Patients Receiving Immune Checkpoint Inhibition. J. Immunother. Cancer 8, e000842. 10.1136/jitc-2020-000842 32581042 PMC7312339

[B54] Laumet G. Edralin J. D. Dantzer R. Heijnen C. J. Kavelaars A. (2019). Cisplatin Educates CD8+ T Cells to Prevent and Resolve Chemotherapy-Induced Peripheral Neuropathy in Mice. Pain 160, 1459–1468. 10.1097/j.pain.0000000000001512 30720585 PMC6527475

[B55] Legakis L. P. Diester C. M. Townsend E. A. Karim-Nejad L. Negus S. S. (2020). Comparison of Chemotherapy Effects on Mechanical Sensitivity and Food-Maintained Operant Responding in Male and Female Rats. Behav. Pharmacol. 31, 477–490. 10.1097/FBP.0000000000000527 31833969 PMC7673225

[B56] Leppert W. Buss T. (2012). The Role of Corticosteroids in the Treatment of Pain in Cancer Patients. Curr. Pain Headache Rep. 16, 307–313. 10.1007/s11916-012-0273-z 22644902 PMC3395343

[B57] Lessans S. Lassiter C. B. Carozzi V. Heindel P. Semperboni S. Oggioni N. (2019). Global Transcriptomic Profile of Dorsal Root Ganglion and Physiological Correlates of Cisplatin-Induced Peripheral Neuropathy. Nurs. Res. 68, 145–155. 10.1097/NNR.0000000000000338 30586060

[B58] Levi S. Taveggia C. (2014). Iron Homeostasis in Peripheral Nervous System, Still a Black Box? Antioxid. Redox Signal 21, 634–648. 10.1089/ars.2013.5813 24409826 PMC4085993

[B59] Li D. Kim W. Shin D. Jung Y. Bae H. Kim S. K. (2016). Preventive Effects of Bee Venom Derived Phospholipase A₂ on Oxaliplatin-Induced Neuropathic Pain in Mice. Toxins (Basel) 8, 27. 10.3390/toxins8010027 PMC4728549 26797636

[B60] Liu X. Tonello R. Ling Y. Gao Y. J. Berta T. (2019). Paclitaxel-activated Astrocytes Produce Mechanical Allodynia in Mice by Releasing Tumor Necrosis Factor-α and Stromal-Derived Cell Factor 1. J. Neuroinflammation 16, 209–214. 10.1186/s12974-019-1619-9 31707979 PMC6842526

[B61] Lu Q. Krull K. R. Leisenring W. Owen J. E. Kawashima T. Tsao J. C. (2011). Pain in Long-Term Adult Survivors of Childhood Cancers and Their Siblings: a Report from the Childhood Cancer Survivor Study. Pain 152, 2616–2624. 10.1016/j.pain.2011.08.006 21907493 PMC3304496

[B62] Ludman T. Melemedjian O. K. (2019). Bortezomib-induced Aerobic Glycolysis Contributes to Chemotherapy-Induced Painful Peripheral Neuropathy. Mol. Pain 15, 1744806919837429. 10.1177/1744806919837429 30810076 PMC6452581

[B63] Luo X. Huh Y. Bang S. He Q. Zhang L. Matsuda M. (2019). Macrophage Toll-like Receptor 9 Contributes to Chemotherapy-Induced Neuropathic Pain in Male Mice. J. Neurosci. 39, 6848–6864. 10.1523/JNEUROSCI.3257-18.2019 31270160 PMC6733562

[B64] Ma X. Chen Y. Li X.-C. Mi W.-L. Chu Y.-X. Wang Y.-Q. (2022). Spinal Neuronal GRK2 Contributes to Preventive Effect by Electroacupuncture on Cisplatin-Induced Peripheral Neuropathy in Mice. Anesth. Analgesia 134, 204–215. 10.1213/ANE.0000000000005768 PMC8647702 34652301

[B65] Maddams J. Utley M. Møller H. (2012). Projections of Cancer Prevalence in the United Kingdom, 2010-2040. Br. J. Cancer 107, 1195–1202. 10.1038/bjc.2012.366 22892390 PMC3461160

[B66] Madu A. J. Ughasoro M. D. (2017). Anaemia of Chronic Disease: An In-Depth Review. Med. Princ. Pract. 26, 1–9. 10.1159/000452104 27756061 PMC5588399

[B67] Maggio M. Guralnik J. M. Longo D. L. Ferrucci L. (2006). Interleukin-6 in Aging and Chronic Disease: A Magnificent Pathway. J. Gerontol. A Biol. Sci. Med. Sci. 61, 575–584. 10.1093/gerona/61.6.575 16799139 PMC2645627

[B68] Maihöfner C. Diel I. Tesch H. Quandel T. Baron R. (2021). Chemotherapy-induced Peripheral Neuropathy (CIPN): Current Therapies and Topical Treatment Option with High-Concentration Capsaicin. Support Care Cancer 29, 4223–4238. 10.1007/s00520-021-06042-x 33624117 PMC8236465

[B69] Makker P. G. Duffy S. S. Lees J. G. Perera C. J. Tonkin R. S. Butovsky O. (2017). Characterisation of Immune and Neuroinflammatory Changes Associated with Chemotherapy-Induced Peripheral Neuropathy. PLOS ONE 12, e0170814. 10.1371/journal.pone.0170814 28125674 PMC5268425

[B70] Marmiroli P. Riva B. Pozzi E. Ballarini E. Lim D. Chiorazzi A. (2017). Susceptibility of Different Mouse Strains to Oxaliplatin Peripheral Neurotoxicity: Phenotypic and Genotypic Insights. PLoS ONE 12, e0186250–25. 10.1371/journal.pone.0186250 29020118 PMC5636145

[B71] McDonald E. S. Randon K. R. Knight A. Windebank A. J. (2005). Cisplatin Preferentially Binds to DNA in Dorsal Root Ganglion Neurons *In Vitro* and *In Vivo*: a Potential Mechanism for Neurotoxicity. Neurobiol. Dis. 18, 305–313. 10.1016/j.nbd.2004.09.013 15686959

[B72] McKelvey R. Berta T. Old E. Ji R. R. Fitzgerald M. (2015). Neuropathic Pain Is Constitutively Suppressed in Early Life by Anti-inflammatory Neuroimmune Regulation. J. Neurosci. 35, 457–466. 10.1523/JNEUROSCI.2315-14.2015 25589741 PMC4293402

[B73] McSweeney K. R. Gadanec L. K. Qaradakhi T. Ali B. A. Zulli A. Apostolopoulos V. (2021). Mechanisms of Cisplatin-Induced Acute Kidney Injury: Pathological Mechanisms, Pharmacological Interventions, and Genetic Mitigations. Cancers 13, 1572. 10.3390/cancers13071572 33805488 PMC8036620

[B74] Meijer C. de Vries E. G. Marmiroli P. Tredici G. Frattola L. Cavaletti G. (1999). Cisplatin-induced DNA-Platination in Experimental Dorsal Root Ganglia Neuronopathy. Neurotoxicology 20, 883–887. 10693969

[B75] Miyagi M. Y. Seelaender M. Castoldi A. de Almeida D. C. Bacurau A. V. Andrade-Oliveira V. (2014). Long-term Aerobic Exercise Protects against Cisplatin-Induced Nephrotoxicity by Modulating the Expression of IL-6 and HO-1. PLoS One 9, e108543. 10.1371/journal.pone.0108543 25272046 PMC4182716

[B76] Montague K. Simeoli R. Valente J. Malcangio M. (2018). A Novel Interaction between CX3CR1 and CCR2 Signalling in Monocytes Constitutes an Underlying Mechanism for Persistent Vincristine-Induced Pain. J. Neuroinflammation 15. 10.1186/s12974-018-1116-6 PMC5889528 29625610

[B77] Montague-Cardoso K. Pitcher T. Chisolm K. Salera G. Lindstrom E. Hewitt E. (2020). Changes in Vascular Permeability in the Spinal Cord Contribute to Chemotherapy-Induced Neuropathic Pain. Brain Behav. Immun. 83, 248–259. 10.1016/j.bbi.2019.10.018 31669344 PMC6928576

[B78] Muckenthaler M. U. Rivella S. Hentze M. W. Galy B. (2017). A Red Carpet for Iron Metabolism. Cell. 168, 344–361. 10.1016/j.cell.2016.12.034 28129536 PMC5706455

[B79] Nozaki Y. Kinoshita K. Hino S. Yano T. Niki K. Hirooka Y. (2015). Signaling Rho-Kinase Mediates Inflammation and Apoptosis in T Cells and Renal Tubules in Cisplatin Nephrotoxicity. Am. J. Physiol. Ren. Physiol. 308, F899–F909. 10.1152/ajprenal.00362.2014 25651561

[B80] Nuver J. de Haas E. C. van Zweeden M. Gietema J. A. Meijer C. (2010). Vascular Damage in Testicular Cancer Patients: a Study on Endothelial Activation by Bleomycin and Cisplatin *In Vitro* . Oncol. Rep. 23, 247–253. 19956889

[B81] Old E. A. Nadkarni S. Grist J. Gentry C. Bevan S. Kim K. W. (2014). Monocytes Expressing CX3CR1 Orchestrate the Development of Vincristine-Induced Pain. J. Clin. Invest. 124, 2023–2036. 10.1172/JCI71389 24743146 PMC4001538

[B82] Onk D. Mammadov R. Suleyman B. Cimen F. K. Cankaya M. Gul V. (2018). The Effect of Thiamine and its Metabolites on Peripheral Neuropathic Pain Induced by Cisplatin in Rats. Exp. Anim. 67, 259–269. 10.1538/expanim.17-0090 29332858 PMC5955757

[B83] Paice J. A. (2011). Chronic Treatment-Related Pain in Cancer Survivors. Pain 152, S84–S89. 10.1016/j.pain.2010.10.010 21036475

[B84] Paice J. A. (2009). Clinical Challenges: Chemotherapy-Induced Peripheral Neuropathy. Semin. Oncol. Nurs. 25, S8–S19. 10.1016/j.soncn.2009.03.013 19447319

[B85] Paice J. A. (2019). Pain in Cancer Survivors: How to Manage. Curr. Treat. Options Oncol. 20, 48. 10.1007/s11864-019-0647-0 31062182

[B86] Piperno A. Pelucchi S. Mariani R. (2020). Inherited Iron Overload Disorders. Transl. Gastroenterol. Hepatol. 5, 25. 10.21037/tgh.2019.11.15 32258529 PMC7063521

[B87] Podratz J. L. Knight A. M. Ta L. E. Staff N. P. Gass J. M. Genelin K. (2011). Cisplatin Induced Mitochondrial DNA Damage in Dorsal Root Ganglion Neurons. Neurobiol. Dis. 41, 661–668. 10.1016/j.nbd.2010.11.017 21145397 PMC3031677

[B88] Portilla D. Li S. Nagothu K. K. Megyesi J. Kaissling B. Schnackenberg L. (2006). Metabolomic Study of Cisplatin-Induced Nephrotoxicity. Kidney Int. 69, 2194–2204. 10.1038/sj.ki.5000433 16672910

[B89] Poulsen J. N. Larsen F. Duroux M. Gazerani P. (2014). Primary Culture of Trigeminal Satellite Glial Cells: a Cell-Based Platform to Study Morphology and Function of Peripheral Glia. Int. J. Physiol. Pathophysiol. Pharmacol. 6, 1–12. 24665354 PMC3961097

[B90] Prentice A. M. (2017). Clinical Implications of New Insights into Hepcidin-Mediated Regulation of Iron Absorption and Metabolism. Ann. Nutr. Metab. 71 Suppl 3, 40–48. 10.1159/000480743 29268258

[B91] Quaresma M. Coleman M. P. Rachet B. (2015). 40-year Trends in an Index of Survival for All Cancers Combined and Survival Adjusted for Age and Sex for Each Cancer in England and Wales, 1971-2011: A Population-Based Study. Lancet 385, 1206–1218. 10.1016/S0140-6736(14)61396-9 25479696

[B92] Ramesh G. Zhang B. Uematsu S. Akira S. Reeves W. B. (2007). Endotoxin and Cisplatin Synergistically Induce Renal Dysfunction and Cytokine Production in Mice. Am. J. Physiol. Ren. Physiol. 293, F325–F332. 10.1152/ajprenal.00158.2007 17494092

[B93] Ravichandran K. Holditch S. Brown C. N. Wang Q. Ozkok A. Weiser-Evans M. C. (2017). IL-33 Deficiency Slows Cancer Growth but Does Not Protect against Cisplatin-Induced AKI in Mice with Cancer. Am. J. Physiol. Ren. Physiol. 314, F356–F366. 10.1152/ajprenal.00040.2017 PMC5899219 29070568

[B94] Saika F. Matsuzaki S. Kobayashi D. Ideguchi Y. Nakamura T. Y. Kishioka S. (2020). Chemogenetic Regulation of CX3CR1-Expressing Microglia Using Gi-DREADD Exerts Sex-dependent Anti-allodynic Effects in Mouse Models of Neuropathic Pain. Front. Pharmacol. 11, 1–10. 10.3389/fphar.2020.00925 32636748 PMC7318895

[B95] Schappacher K. A. Styczynski L. Baccei M. L. (2017). Early Life Vincristine Exposure Evokes Mechanical Pain Hypersensitivity in the Developing Rat. Pain 158, 1647–1655. 10.1097/j.pain.0000000000000953 28722694 PMC5643007

[B96] Sekijima T. Tanabe A. Maruoka R. Fujishiro N. Yu S. Fujiwara S. (2011). Impact of Platinum-Based Chemotherapy on the Progression of Atherosclerosis. Climacteric 14, 31–40. 10.3109/13697137.2010.522278 21067421

[B97] Seretny M. Currie G. L. Sena E. S. Ramnarine S. Grant R. MacLeod M. R. (2014). Incidence, Prevalence, and Predictors of Chemotherapy-Induced Peripheral Neuropathy: A Systematic Review and Meta-Analysis. Pain 155, 2461–2470. 10.1016/j.pain.2014.09.020 25261162

[B98] Shim H. S. Bae C. Wang J. Lee K. H. Hankerd K. M. Kim H. K. (2019). Peripheral and Central Oxidative Stress in Chemotherapy-Induced Neuropathic Pain. Mol. Pain 15, 1744806919840098. 10.1177/1744806919840098 30857460 PMC6458664

[B99] Staff N. P. Cavaletti G. Islam B. Lustberg M. Psimaras D. Tamburin S. (2019). Platinum-induced Peripheral Neurotoxicity: From Pathogenesis to Treatment. J. Peripher Nerv. Syst. 24 Suppl 2, S26. 10.1111/jns.12335 31647151 PMC6818741

[B100] Starobova H. Mueller A. Allavena R. Lohman R. J. Sweet M. J. Vetter I. (2019). Minocycline Prevents the Development of Mechanical Allodynia in Mouse Models of Vincristine-Induced Peripheral Neuropathy. Front. Neurosci. 13, 1–10. 10.3389/fnins.2019.00653 31316337 PMC6610325

[B101] Starobova H. Mueller A. Deuis J. R. Carter D. A. Vetter I. (2020). Inflammatory and Neuropathic Gene Expression Signatures of Chemotherapy-Induced Neuropathy Induced by Vincristine, Cisplatin, and Oxaliplatin in C57BL/6J Mice. J. Pain 21, 182–194. 10.1016/j.jpain.2019.06.008 31260808

[B102] Summers S. A. Chan J. Gan P. Y. Dewage L. Nozaki Y. Steinmetz O. M. (2011). Mast Cells Mediate Acute Kidney Injury through the Production of TNF. J. Am. Soc. Nephrol. 22, 2226–2236. 10.1681/ASN.2011020182 22021718 PMC3279934

[B103] Ta L. E. Espeset L. Podratz J. Windebank A. J. (2006). Neurotoxicity of Oxaliplatin and Cisplatin for Dorsal Root Ganglion Neurons Correlates with Platinum-DNA Binding. Neurotoxicology 27, 992–1002. 10.1016/j.neuro.2006.04.010 16797073

[B104] Tanner K. D. Reichling D. B. levine J. D. (1998). Nociceptor Hyper-Responsiveness during Vincristine-Induced Painful Peripheral Neuropathy in the Rat. J. Neurosci. 18, 6480–6491. 10.1523/jneurosci.18-16-06480.1998 9698336 PMC6793188

[B105] Vela D. (2018). The Dual Role of Hepcidin in Brain Iron Load and Inflammation. Front. Neurosci. 12, 740. 10.3389/fnins.2018.00740 30374287 PMC6196657

[B106] Vencappa S. Donaldson L. F. Hulse R. P. (2015). Cisplatin Induced Sensory Neuropathy Is Prevented by Vascular Endothelial Growth Factor-A. Am. J. Transl. Res. 7, 1032–1044. 26279748 PMC4532737

[B107] Vichaya E. G. Chiu G. S. Krukowski K. Lacourt T. E. Kavelaars A. Dantzer R. (2015). Mechanisms of Chemotherapy-Induced Behavioral Toxicities. Front. Neurosci. 9, 1–10. 10.3389/fnins.2015.00131 25954147 PMC4404721

[B108] Walter P. B. Knutson M. D. Paler-Martinez A. Lee S. Xu Y. Viteri F. E. (2002). Iron Deficiency and Iron Excess Damage Mitochondria and Mitochondrial DNA in Rats. Proc. Natl. Acad. Sci. U. S. A. 99, 2264–2269. 10.1073/pnas.261708798 11854522 PMC122353

[B109] Wan C. F. Zheng L. L. Liu Y. Yu X. (2016). Houttuynia Cordata Thunb Reverses Oxaliplatin-Induced Neuropathic Pain in Rat by Regulating Th17/Treg Balance. Am. J. Transl. Res. 8, 1609–1614. 27186286 PMC4859645

[B110] Wang X. M. Hamza M. Wu T. X. Dionne R. A. (2009). Upregulation of IL-6, IL-8 and CCL2 Gene Expression after Acute Inflammation: Correlation to Clinical Pain. Pain 142, 275–283. 10.1016/j.pain.2009.02.001 19233564 PMC3513699

[B111] West A. P. Koblansky A. A. Ghosh S. (2006). Recognition and Signaling by Toll-like Receptors. Annu. Rev. Cell. Dev. Biol. 22, 409–437. 10.1146/annurev.cellbio.21.122303.115827 16822173

[B112] Winston J. A. Safirstein R. (1985). Reduced Renal Blood Flow in Early Cisplatin-Induced Acute Renal Failure in the Rat. Am. J. Physiol. 249, F490–F496. 10.1152/ajprenal.1985.249.4.F490 4051003

[B113] Yu M. Han J. Cui P. Dai M. Li H. Zhang J. (2008). Cisplatin Up-Regulates ICAM-1 Expression in Endothelial Cell via a NF-kappaB Dependent Pathway. Cancer Sci. 99, 391–397. 10.1111/j.1349-7006.2008.00696.x 18271937 PMC11159323

[B114] Zhang B. Ramesh G. Uematsu S. Akira S. Reeves W. B. (2008). TLR4 Signaling Mediates Inflammation and Tissue Injury in Nephrotoxicity. J. Am. Soc. Nephrol. 19, 923–932. 10.1681/ASN.2007090982 18256356 PMC2386719

[B115] Zhang H. Li Y. de Carvalho-Barbosa M. Kavelaars A. Heijnen C. J. Albrecht P. J. (2016). Dorsal Root Ganglion Infiltration by Macrophages Contributes to Paclitaxel Chemotherapy-Induced Peripheral Neuropathy. J. Pain 17, 775–786. 10.1016/j.jpain.2016.02.011 26979998 PMC4939513

